# Personalised Long‐Term Albumin Treatment Based on Three‐Month Ascites Response in Patients With Decompensated Cirrhosis

**DOI:** 10.1111/liv.70598

**Published:** 2026-03-18

**Authors:** Enrico Pompili, Giulia Iannone, Salvatore Piano, Pierluigi Toniutto, Antonio Lombardo, Stefania Gioia, Marta Tonon, Roberta Gagliardi, Daniele Carrello, Greta Tedesco, Vito Di Marco, Giacomo Zaccherini, Lorenzo Lani, Maurizio Baldassarre, Silvia Nardelli, Davide Bitetto, Vincenza Calvaruso, Paolo Angeli, Paolo Caraceni

**Affiliations:** ^1^ Department of Medical and Surgical Sciences Alma Mater Studiorum University of Bologna Bologna Italy; ^2^ Unit of Semeiotics, Liver and Alcohol‐Related Diseases IRCCS Azienda Ospedaliero‐Universitaria di Bologna Bologna Italy; ^3^ Unit of Internal Medicine and Hepatology, Department of Medicine–DIMED University and Hospital of Padova Padua Italy; ^4^ Hepatology and Liver Transplantation Unit University Academic Hospital Udine Italy; ^5^ UOC Di Gastroenterologia, Dipartimento Di Promozione Della Salute, Materno Infantile, Medicina Interna e Specialistica (PROMISE) University of Palermo Palermo Italy; ^6^ Department of Translational and Precision Medicine Sapienza University of Rome Rome Italy

**Keywords:** ascites, paracentesis, portal hypertension, refractory ascites, TIPS

## Abstract

**Background and Aims:**

Long‐term albumin (LTA) is effective for treating ascites in decompensated cirrhosis. This study aims to analyse the clinical courses of patients receiving LTA and provide a 3 month stratification to personalise management integrating LTA with other options.

**Methods:**

Patients receiving LTA included in the multicentre, retrospective, observational Real‐ANSWER study were stratified into three categories according to the response of ascites after 3 months of treatment: ‘responders’ (grade 0–1 ascites), ‘partial responders’ (at least grade 2 ascites not receiving therapeutic paracentesis) and ‘non‐responders’ (at least grade 2 ascites receiving therapeutic paracentesis). Clinical trajectories and outcomes of the different categories were compared.

**Results:**

Of the 252 patients included (median Child‐Pugh 9, MELDNa 18), 36% were responders, 29% partial responders and 35% non‐responders. Responders differed significantly from the other groups, with higher cumulative incidence of LTA discontinuation for clinical improvement (33%) and transplantation (26%), a lower 18 month mortality (13%) and minimal use of TIPS. Partial and non‐responders showed similar trajectories with high mortality (35% and 42%) and low incidence of transplantation (12% and 11%). TIPS was performed predominantly among non‐responders (15%). Both groups had a few patients (12% and 8%) able to stop LTA for clinical improvement frequently related to an effective etiologic treatment.

**Conclusions:**

Using a 3 month stratification according to the ascites response of LTA, patients can be grouped into three categories with different clinical courses and outcomes. This may help to stratify prognosis and inform clinical discussions on the management of ascites by integrating LTA with other available options.

## Lay Summary

- Long‐term albumin should be considered an additional weapon on top of diuretics for treatment of clinically evident grade 2 and 3 ascites in patients with decompensated cirrhosis.
- The response of ascites after 3 months of long‐term albumin (responders, partial responders and non‐responders) stratifies patients with different trajectories and outcomes.
- This stratification may help to guide long‐term albumin treatment at the individual level and integrate this approach with the other options available for the management of difficult‐to‐treat ascites.

**Abbreviations**
ACLF: Acute‐on‐Chronic Liver Failure
CI: confidence interval
EASL: European Association for the Study of the Liver
HA: human albumin
Hb: haemoglobin
HCC: hepatocellular carcinoma
HE: hepatic encephalopathy
ICA: International Club of Ascites
INR: International Normalised Ratio
IQR: Interquartile range
LT: Liver Transplantation
LTA: long‐term albumin
MASLD: metabolic dysfunction‐associated steatotic liver disease
MELD: Model for End‐stage Liver disease
MELD‐Na: Model for End‐stage Liver disease incorporating serum sodium
NSBB: non‐selective beta‐blockers
RCT: randomised clinical trial
SBP: spontaneous bacterial peritonitis
TIPS: Transjugular intrahepatic portosystemic shunt
WBC: white blood cells

Human albumin (HA) is frequently used to prevent or treat acute complications of cirrhosis [1]. Over the last decade, long‐term albumin (LTA) has been proposed for the management of ascites [2]. However, randomised controlled trials have produced conflicting results [3, 4, 5], suggesting that the efficacy of LTA is not universal and that a better definition of patients who can receive benefit from this treatment is needed.

Following the publication of the ANSWER trial [3], LTA was incorporated into the Italian Association for the Study of the Liver (AISF) recommendations as a treatment option for patients with ascites, resulting in the regular use in many hepatology centres in Italy [6]. In recent years, the use of LTA has expanded beyond Italy, with real‐world experience from Australia, India and Spain reporting improved ascites control in routine practice [7, 8, 9]. Furthermore, the Asia‐Pacific Association for Study of Liver (APASL) and Chinese guidelines consider adding albumin in the management of ascites [10, 11].

Rather than an alternative to existing therapies, LTA should be viewed as an additional tool within the comprehensive management of ascites. In this regard, the recent EASL clinical practice guidelines recommend early discussion of transjugular intrahepatic portosystemic shunt (TIPS) in patients who continue to require large‐volume paracentesis (LVP) despite optimised medical therapy^12^. Because evidence is limited, neither a minimum number of LVPs nor an optimal observation window can be firmly recommended [12].

A multicentre retrospective study (Real‐ANSWER) recently reported on the real‐world experience of LTA at five Italian centres. In this cohort, ascites improved to grade 0–1 in more than half of the patients, with approximately one‐third improving within 3 months and most of these maintaining their response thereafter [13].

Building on those observations, we hypothesised that a simple assessment of ascites response at 3 months could delineate distinct post‐landmark clinical trajectories and contribute to treatment decision. We therefore performed a post hoc analysis of the Real‐ANSWER cohort using a prespecified 3 month landmark to classify patients by ascites response, describe subsequent outcomes and translate these findings into a practical, hypothesis‐generating conceptual framework that integrates LTA within comprehensive care for patients with cirrhosis and ascites.

## 1 PATIENTS and METHODS

### 1.1 Study Design

This study is based on the Real‐ANSWER cohort, a multicentre, retrospective, observational study of patients with cirrhosis and ascites treated with LTA in five Italian tertiary hepatology centres [13]. Detailed information on the inclusion and exclusion criteria can be found in the Supporting Information. Briefly, patients with cirrhosis and ascites who started LTA between January 2016 and February 2022 were included in the study. TIPS before starting LTA was an exclusion criterion. The time of inclusion corresponds to the time of initiation of LTA.

Most patients received a median dose of 40 g of albumin (IQR 40–40), administered once weekly, either in outpatient clinics or through territorial healthcare services. Further details regarding the modalities of LTA treatment can be found elsewhere [6, 13]. During the study period, besides LTA, HA was also given for the indications supported by International guidelines [14].

For the purposes of the present study, we excluded patients with grade 1 ascites at enrolment, for whom LTA is not recommended by AISF guidelines [6], and those who received LTA for less than 3 months. All patients were followed until death, liver transplantation (LT) or up to a maximum of 18 months of follow‐up.

The study protocol conforms to the ethical guidelines of the 1975 Declaration of Helsinki and has been approved by the local ethical committees at each participating centre.

### 1.2 Data Collection

At the time of enrolment, demographic and anthropometric data, clinical history, and clinical and laboratory data were collected. Clinical and laboratory data were also recorded when available during follow‐up at 1, 3 and then every 3 months from inclusion. The incidence of LTA discontinuation, including its cause, death, LT or TIPS placement, was recorded up to 18 months.

### 1.3 Definition and Classification

We used a prespecified 3 month landmark design to classify patients based on ascites response. Patients were classified as: (i) ‘responders’, presenting with grade 0–1 ascites after 3 months of LTA; (ii) ‘partial responders’: showing at least grade 2 ascites and not receiving therapeutic paracentesis in the second or third months of LTA; (iii) ‘non‐responders’: showing at least grade 2 ascites requiring one or more therapeutic paracenteses in the second or third months of LTA. Grade of ascites and refractory ascites have been defined according to the EASL guidelines^14^. Ascites grading was performed locally at each centre by experienced hepatologists.

Patients were further stratified at baseline into favourable, intermediate and unfavourable benefit/risk categories for TIPS placement in accordance with EASL guidelines [12]. Specifically, patients with none of the following risk factors were classified as ‘low risk’: age < 65 years; no history of hepatic encephalopathy (HE); Child‐Pugh score < 10; MELD score < 12; absence of cardiovascular disease. Patients presenting with at least one high‐risk criterion (Child‐Pugh score > 12, MELD score > 18, or age > 70) were categorised as ‘high risk’. Those presenting with at least one intermediate‐risk criterion (Child‐Pugh score 10–12, MELD score 12–18, or ages 65–70) were classified accordingly. Due to the retrospective nature of the current study (which was not originally designed for this analysis) and the difficulty in distinguishing between ‘previous overt HE’ and ‘recurrent HE’, as well as between moderate and severe cardiac dysfunction, a pragmatic approach was adopted. The 33 patients who met one of these two criteria, but who had no other high‐risk features (i.e., a Child–Pugh score of > 12, a MELD score of > 18 or an age of > 70), were classified as ‘intermediate‐risk’.

### 1.4 Statistical Analysis

For all continuous parameters the normality of distribution and homogeneity of variance were evaluated by the Shapiro–Wilk and Levene tests, then variables were reported as mean and standard deviation or median and interquartile range (IQR) as appropriate. Accordingly, comparisons between groups were performed using the Student's *t*‐test, the Mann–Whitney U test or the analysis of variance (ANOVA) when appropriate. In the latter case, correction for multiple comparisons was performed using the Bonferroni method. Categorical parameters were reported as frequency and percentage and compared using the chi‐square or Fisher's exact test.

Cumulative incidence functions (CIFs) for LTA discontinuation by reason (clinical improvement, death, LT, TIPS, other) were estimated treating alternative reasons as competing events. CIFs for 18 month mortality considered LT as a competing event. The time origin for all post‐landmark analyses (CIFs and regressions) was set at 3 months after LTA initiation. To assess potential selection bias from excluding patients with LTA duration < 3 months, we compared baseline characteristics between included patients and those excluded for < 3 months of LTA and described reasons for early discontinuation in the excluded group (Table S1). CIFs were computed using the ‘stcompet’ command in Stata version 18 [15], and compared using Gray's test. Predictors of 18 month mortality were assessed using multivariable Fine‐Gray models (LT as a competing event). A backward stepwise elimination was employed to identify a parsimonious set of predictors, specifically aiming to evaluate the independent prognostic value of the categories of response to LTA at 3 months (responders, partial responders and non‐responders) compared to established clinical parameters. Variables entered into multivariable models were selected from those significantly associated to mortality (*p* < 0.05) at univariable analysis.

To investigate the difference in rate of LT, a Propensity Score Matching (PSM) analysis was conducted. Responders were matched 1:1 with Partial/Non‐Responders based on age, MELD score, grade of ascites and extrahepatic comorbidities (calliper 0.2). Balance was assessed using standardised bias and *t*‐tests. In the matched cohort, outcomes were compared using Fine‐Gray competing risk models.

All tests were two‐sided and values of *p* less than 0.05 were considered statistically significant. Statistical analysis was performed using STATA (StataCorp LLC, College Station, TX, USA) version 18.

## 2 Results

### 2.1 Patient Disposition

**FIGURE 1:**
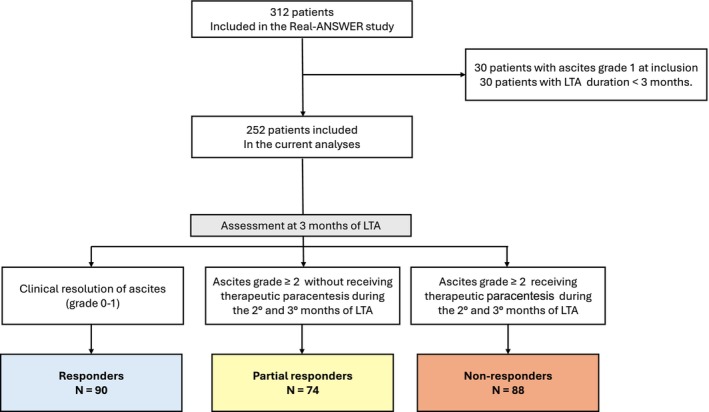
Patient flow diagram. LTA, Long‐term albumin.

Three‐hundred and twelve patients were included in the Real‐ANSWER cohort. As detailed in the Methods section, for the purposes of this analysis, we excluded 30 patients with grade 1 ascites at inclusion and 30 patients who had received LTA for less than 3 months (Figure 1). Therefore, the current analysis included a study population of 252 patients. To mitigate the risk of selection bias, we compared the baseline characteristics of patients who were included with those who were excluded due to LTA duration being less than 3 months (*n* = 30), and no statistical differences were observed (see Table S1). The reasons for LTA discontinuation in the latter group are shown in (Table S2). After 3 months of LTA, 90 patients (36%) were responders, 74 (29%) were partial responders and 88 (35%) were non‐responders (Figure 1).

### 2.2 Baseline Characteristics According to Ascites Response at 3 Months

The baseline characteristics (at the time of starting LTA) of the 3 groups of patients are reported in (Table 1). Briefly, responders were younger, while ascites was significantly more severe in non‐responder patients who had a higher prevalence of grade 3 or refractory ascites and need for therapeutic paracentesis prior to enrolment as compared to the other 2 groups. Aetiology of cirrhosis, medical history, extrahepatic comorbidities and drug therapies were similar among the three groups.

**TABLE 1:** Baseline characteristics of patients included in the study according to ascites response after 3 months of long‐term albumin treatment.

	Responders *N* = 90	Partial responders *N* = 74	Non‐responders *N* = 88	*p*
Demographic data				
Age (years)	60 (52–68)^a^	64 (54–72)	64 (58–70)	0.022
Male sex (*n*, %)	60 (67)	48 (65)	67 (76)	0.233
Aetiology of cirrhosis				0.375
Alcohol (*n*, %)	42 (47)	21 (28)	36 (41)	
MASLD (*n*, %)	11 (12)	12 (16)	14 (16)	
Viral (*n*, %)	11 (12)	10 (14)	12 (14)	
Alcohol + viral (*n*, %)	11 (12)	10 (14)	6 (7)	
Alcohol + MASLD (*n*, %)	8 (9)	12 (16)	9 (10)	
Other (*n*, %)	7 (8)	9 (12)	11 (13)	
Ascites				
Ascites grade				0.001
Ascites grade 2 (*n*, %)	66 (73)	57 (77)	30 (34)	
Ascites grade 3 (*n*, %)	24 (27)	17 (23)	58 (66)	
Refractory ascites (*n*, %)	7 (8)	13 (18)	52 (59)	0.001
Previous paracentesis within 6 months prior to enrolment (*n*, %)	27 (30)	32 (44)	70 (79)	0.001
1–3 paracentesis in last 6 months	24 (27)	24 (32)	25 (28)	
≥ 4 paracenteses in last 6 months	3 (3)	8 (11)	45 (51)	
Medical History				
Presence of oesophageal varices (*n*, %)	73 (81)	50 (68)	66 (75)	0.137
Previous overt HE (*n*, %)	23 (26)	22 (30)	24 (27)	0.837
Previous gastrointestinal bleeding (*n*, %)	11 (12)	15 (20)	18 (21)	0.264
Previous spontaneous bacterial peritonitis (*n*, %)	10 (11)	10 (14)	9 (10)	0.799
Previous hepatorenal syndrome (*n*, %)	9 (10)	6 (8)	6 (7)	0.766
HCC within Milan criteria (*n*, %)	9 (10)	10 (14)	7 (8)	0.507
Active alcohol consumption (*n*, %)	21 (23)	9 (12)	10 (11)	0.054
Etiological treatment (*n*, %) (< 12 month before or during LTA)	40 (44)	31 (42)	26 (30)	0.096
Medications				
Anti‐aldosteronic drugs (mg/day)	200 (100–300)	200 (100–300)	200 (100–200)	0.659
Furosemide (mg/day)	50 (25–75)	50 (25–75)	50 (25–75)	0.374
NSBB (*n*, %)	49 (54)	37 (50)	42 (48)	0.660
Rifaximin for HE prophylaxis (*n*, %)	39 (43)	23 (31)	33 (38)	0.273
Comorbidities				
Chronic Heart Disease (*n*, %)	13 (14)	14 (19)	16 (18)	0.707
Chronic kidney disease (*n*, %)	11 (12)	8 (11)	20 (23)	0.068
Chronic lung disease (*n*, %)	10 (11)	4 (5)	11 (13)	0.289
Diabetes (*n*, %)	26 (29)	24 (32)	34 (39)	0.379
Laboratory and hemodynamic data at inclusion				
Bilirubin (mg/dL)	2.5 (1.5–4.7)	1.8 (1.1–3.3)	1.7 (1.1–3.7)	0.017
Albumin (g/L)	30.7 (27.0–34.0)	31.0 (27.0–34.0)	31.5 (28.5–35.0)	0.259
INR	1.5 (1.3–1.7)^b^	1.3 (1.2–1.6)	1.3 (1.2–1.5)	< 0.001
Creatinine (mg/dL)	0.9 (0.7–1.1)^c^	0.9 (0.8–1.2)	1.1 (0.8–1.4)	0.004
Sodium (mmol/L)	136 (134–138)	136 (134–139)	135 (132–139)	0.276
WBC (10^9^/L)	5.0 (3.6–7.5)	4.8 (3.8–6.2)	5.9 (5.0–7.6)	0.008
Haemoglobin (g/dL)	10.8 (9.3–12.2)	10.5 (9.5–12.3)	10.9 (9.6–12.9)	0.627
Prognostic scores				
Child‐Pugh score	9 (8–10)	8 (7–10)	8 (7–10)	0.394
MELD score	15 (13–19)^c^	14 (11–17)	14 (11–18)	0.022
MELD‐Na score	19 (16–22)	17 (13–21)	18 (14–22)	0.140
Benefit/risk stratification for TIPS*				0.400
Low risk/High benefit	9 (10)	10 (14)	15 (17)	
Intermediate risk/Intermediate benefit	47 (52)	32 (43)	34 (39)	
High‐risk/Low‐benefit	34 (38)	32 (43)	39 (44)	

*Note:* Data are reported by median and interquartile range or absolute frequency and percentage (%) as appropriate.

Abbreviations: HCC, hepatocellular carcinoma; HE, hepatic encephalopathy; INR, International Normalised Ratio; MASLD, metabolic dysfunction‐associated steatotic liver disease; MELD, Model for End‐stage Liver disease; MELD‐Na, Model for End‐stage Liver disease incorporating serum sodium; NSBB, non‐selective beta blockers; WBC, white blood cells.

^a^ *p* < 0.05 vs partial responders.

^b^ *p* < 0.05 vs non‐responders.

^c^ *p* < 0.05 vs partial‐ and non‐responders.

* According to the EASL Clinical Practice Guidelines on TIPS.

Regarding laboratory parameters, creatinine and white blood cell count were significantly higher in non‐responders, while INR and bilirubin were slightly but significantly higher in responders. Baseline serum albumin level was about 31 g/L in all the 3 groups. Finally, MELD score was significantly higher in responders compared to partial and non‐responders, although the difference (15 vs. 14) is likely not clinically relevant, while the MELD‐Na and Child‐Pugh scores were similar.

According to the stratification of the benefit/risk balance of TIPS placement proposed by the EASL guidelines [12], only 34 patients (13%) could be considered to have a favourable risk/benefit ratio for TIPS, 113 (45%) an intermediate risk/benefit ratio and 105 (42%) an unfavourable risk/benefit ratio.

### 2.3 Clinical Trajectories According to Ascites Response

**FIGURE 2:**
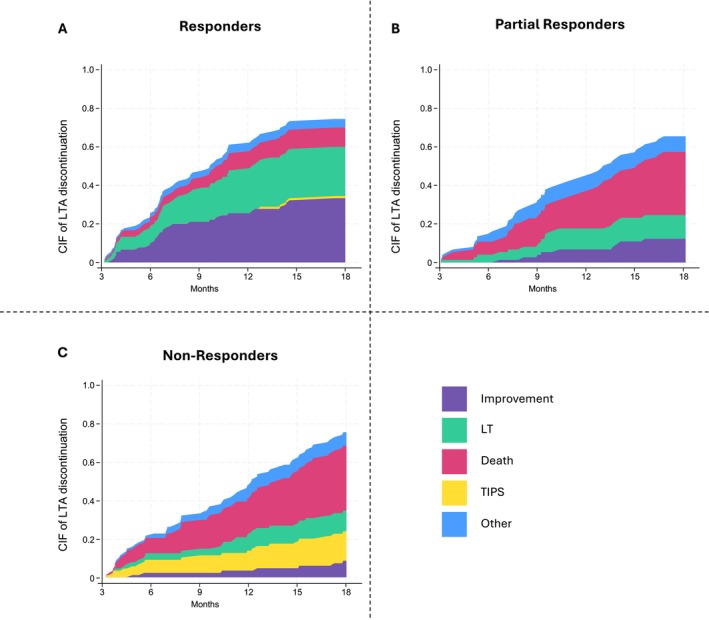
Stacked cumulative incidence curves of LTA discontinuation due to clinical improvement (purple), TIPS placement (yellow), LT (green), death (red) or other causes in the three patient categories: ‘responders’ (panel A), ‘partial responders’ (panel B) and ‘non‐responders’ (panel C). For each line, other causes of LTA discontinuation are treated as competing risk events.

After 18 months from LTA initiation, 77% of patients were no longer on LTA among responders, 65% among partial responders and 72% among non‐responders. Figure 2 shows the CIF of the reasons for LTA discontinuation. Briefly, in the responder group, 33% of patients stopped LTA due to stable clinical improvement, 11% died, 26% received LT and 1% TIPS, and 5% discontinued LTA for other causes including referral to palliative care (Table 2). CIF of death was significantly higher (11% vs. 33% and 34%, respectively, *p* < 0.001) and LT significantly lower in partial and non‐responders (26% vs. 12% and 11%, respectively, *p* = 0.012). In addition, 1 patient received TIPS among the responders, none among the partial responders and 13 patients (15%) among the non‐responders. Interestingly, even in the partial responder and non‐responder groups a small number of patients were able to discontinue LTA due to clinical improvement (12% and 8%, respectively) (Table 2). Of note, more than half of these patients (5 out of 9 in partial responders and 5 out of 7 in non‐responders) had received etiological treatment during the study period or in the preceding 6 months. Overall, 92% of responders, 46% of partial responders and 20% of non‐responders had achieved clinical resolution of ascites at the last observation before discontinuation of LTA for any reason. Interestingly, 14 out of 34 (41%) of patients with a favourable risk/benefit ratio for TIPS were able to discontinue LTA for ascites resolution. On the other hand, 26 patients received the diagnosis of refractory ascites during the entire follow‐up period (excluding those with refractory ascites at the time of inclusion). Of these patients, one was in the ‘responders’ subgroup, 6 were in ‘partial responders’, and 19 were in ‘non‐responders’. Finally, in the subgroup of the 72 patients who were still receiving LTA at 18 months, 19 (90%) of responders, 15 (58%) of partial responders and 10 (40%) of non‐responders had clinical resolution of ascites.

**TABLE 2:** Cumulative incidence of long‐term albumin discontinuation by reason at 6, 12 and 18 months from long‐term albumin initiation.

Reasons for LTA discontinuation	6 month LTA discontinuation CIF and 95% CI	12 month LTA discontinuation CIF and 95% CI	18 month LTA discontinuation CIF and 95% CI	Patients (*n*)
Responders				
Stop for clinical improvement	9% (4–16)	26% (17–35)	33% (24–43)	30
LT	9% (4–16)	22% (14–31)	26% (17–35)	23
TIPS placement	0%	0%	1% (0–5)	1
Death	3% (1–8)	9% (4–16)	11% (5–17)	10
Other	2% (0–7)	4% (1–10)	5% (2–12)	5
Still on LTA at 18 months				21
Partial responders				
Stop for clinical improvement	0%	7% (3–14)	12% (6–21)	9
LT	4% (1–10)	11% (5–19)	12% (6–21)	9
TIPS placement	0%	0%	0%	0
Death	7% (2–14)	19% (11–28)	33% (22–44)	24
Other	3% (1–8)	8% (3–16)	8% (3–16)	6
Still on LTA at 18 months				26
Non‐responders				
Stop for clinical improvement	2% (0–7)	4% (1–9)	8% (4–16)	7
LT	3% (1–9)	8% (4–15)	11% (5–18)	9
TIPS placement	7% (3–13)	10% (5–18)	15% (9–24)	13
Death	8% (4–14)	19% (11–27)	34% (24–44)	28
Other	1% (0–6)	7% (3–14)	7% (3–14)	6
Still on LTA at 18 months				25

*Note:* Time points are measured from LTA initiation (6/12/18 months), which correspond to 3/9/15 months from the 3 month landmark.

Abbreviations: CI, confidence interval; LT, liver transplantation; LTA, Long‐term Albumin; TIPS, Transjugular intrahepatic portosystemic shunt.

To understand why LT was significantly more frequent in the responder compared to the other two groups, we also performed a PSM analysis generating a matched cohort of 170 patients (85 responders and 85 partial/non‐responders) who were balanced for baseline age, MELD score, extrahepatic comorbidities and ascites grade (Table S3, Figure S1). In this matched population, the difference in the cumulative incidence of LT between groups was no longer statistically significant (SHR 1.65, 95% CI 0.80–3.40, *p* = 0.168, Figure S2), indicating that the lower access to LT in the partial/non‐responder groups was probably driven by baseline demographic and clinical characteristics rather than by the ascites response.

### 2.4 Cumulative Incidence of Death

**FIGURE 3:**
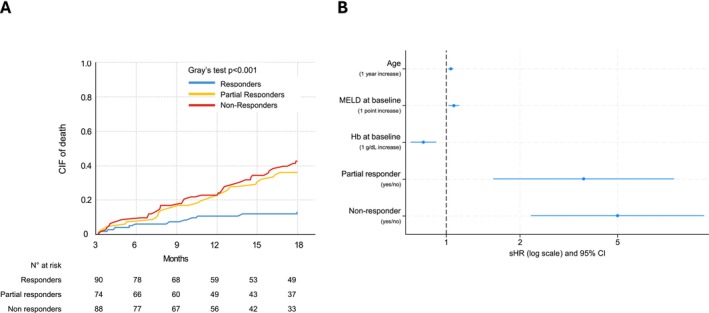
*Panel A*. Cumulative incidence function (CIF) of 18 month mortality in the three categories of patients. Liver transplant (LT) has been considered as a competing event. Blue: Responders; yellow: Partial responders; orange: Non‐responders. *Panel B*. Independent predictors of 18 month mortality. Plot of the multivariable competing risk regression model with stepwise backward elimination according to the Fine and Gray method (liver transplantation was considered as a competing event). Parameters included in the initial model were age, aetiology of cirrhosis, presence of extrahepatic comorbidities (yes/no), history of gastrointestinal bleeding (yes/no), etiological treatment (yes/no), MELD score at baseline, haemoglobin at baseline, categories of response to LTA at 3 months (responders, partial responders and non‐responders). Data are reported as subdistribution hazard ratio (sHR) and 95% confidence interval (CI).

Figure 3 (panel A) shows the 18 month CIF of death in the three groups of patients considering LT as a competing event. A total of 62 patients discontinued LTA because of death (10 in responders, 24 in partial responders and 28 in non‐responders), while an additional 9 patients died after discontinuation of LTA. The 18 month CIF for all‐cause death was 13% [95% CI 7%–20%] in responders, 35% [95% CI 25%–46%] in partial responders and 42% [95% CI 31%–53%] in non‐responders (*p* < 0.001). The cause of death was not available in 18% of patients, while, in the remaining patients, the main cause was bacterial infection (27%), liver failure (17%), acute‐on‐chronic liver failure (ACLF) (8%) and HCC (7%). In 21% of cases, the cause was not related to cirrhosis. Notably, in responders and partial responders, the leading causes of death were non‐SBP infections (31% and 27%, respectively) and extra‐hepatic diseases (42% and 24%), whereas in non‐responders, the mortality was predominantly associated with liver failure (22%) and ACLF (12%). To further explore the driver of the high mortality observed in the partial responder group, which was similar to that observed in non‐responders despite a better control of ascites, we also conducted a specific sensitivity analysis in this subgroup (*n* = 74). The analysis showed that the severity of ascites at baseline was not associated with 18 month mortality (sHR 0.83, 95% CI 0.32–2.18, *p* = 0.71).

Overall, patients who died were significantly older compared to survivors (67 vs. 61%, *p* < 0.001) and presented a higher number of extrahepatic comorbidities (65% vs. 46%, *p* = 0.007). Among lab values at baseline, they had higher serum creatinine (1.1 vs. 0.9 mg/dL, *p* = 0.017) and lower haemoglobin (9.9 vs. 11.0 g/dL, *p* < 0.001), with a higher MELD score (16 vs. 14, *p* = 0.043) (Table 3).

**TABLE 3:** Comparison of clinical and laboratory characteristics of patients deceased versus alive or transplanted at 18 months from inclusion.

	Alive or transplanted *N* = 181	Death *N* = 71	*p*
Demographic data			
Age (years)	61 (53–67)	67 (59–74)	< 0.001
Male sex (n, %)	125 (69)	50 (70)	0.833
Aetiology of cirrhosis			0.022
Alcohol (*n*, %)	76 (42)	23 (32)	
MASLD (*n*, %)	21 (12)	16 (23)	
Viral (*n*, %)	25 (14)	8 (11)	
Alcohol + viral (*n*, %)	24 (13)	3 (4)	
Alcohol + MASLD (*n*, %)	20 (11)	9 (13)	
Other (*n*, %)	15 (8)	12 (17)	
Medical History			
Refractory ascites (*n*, %)	48 (27)	24 (34)	0.250
Previous overt HE (*n*, %)	46 (25)	23 (32)	0.264
Previous gastrointestinal bleeding (*n*, %)	25 (14)	19 (27)	0.015
Previous spontaneous bacterial peritonitis (*n*, %)	23 (13)	6 (9)	0.341
Previous hepato‐renal syndrome (*n*, %)	18 (10)	3 (4)	0.134
HCC within Milan criteria (*n*, %)	21 (12)	5 (7)	0.284
Etiological treatment (< 12 month before or during LTA) (*n*, %)	77 (43)	20 (28)	0.035
Comorbidities			
Any extrahepatic comorbidities (*n*, %)	83 (46)	46 (65)	0.007
Chronic Heart Disease (*n*, %)	26 (14)	17 (24)	0.069
Chronic kidney disease (*n*, %)	20 (11)	19 (27)	0.002
Chronic lung disease (*n*, %)	18 (10)	7 (10)	0.984
Diabetes (*n*, %)	52 (29)	32 (45)	0.013
Laboratory data and prognostic scores at inclusion			
Bilirubin (mg/dL)	2.1 (1.2–3.9)	2.0 (1.3–3.8)	0.984
Albumin (g/L)	31.0 (27.1–35.0)	30.5 (27.0–34.0)	0.382
INR	1.4 (1.2–1.6)	1.4 (1.2–1.6)	0.864
Creatinine (mg/dL)	0.9 (0.8–1.2)	1.1 (0.8–1.5)	0.017
Sodium (mmol/L)	136 (133–139)	136 (134–139)	0.522
WBC (10^9^/L)	5.4 (4.0–7.6)	5.1 (3.9–6.3)	0.106
Haemoglobin (g/dL)	11.0 (9.9–12.8)	9.9 (8.8–11.5)	< 0.001
Child‐Pugh score	8.0 (7.0–10.0)	9.0 (8.0–10.0)	0.225
MELD score	14.0 (11.0–17.0)	16.0 (12.0–18.0)	0.043
MELD‐Na score	18.0 (14.0–21.0)	19.0 (16.0–22.0)	0.168
Categories of response to LTA			< 0.001
Responders (*n*, %)	79 (45)	11 (16)	
Partial responders (*n*, %)	48 (27)	26 (37)	
Non responders (*n*, %)	54 (30)	34 (48)	

*Note:* Data are reported by median and interquartile range or absolute frequency and percentage (%) as appropriate.

Abbreviations: HCC, hepatocellular carcinoma; HE, hepatic encephalopathy; INR, international normalised ratio; MASLD, metabolic dysfunction‐associated steatotic liver disease; MELD, model for end‐stage liver disease; MELD‐Na, model for end‐stage liver disease incorporating serum sodium; NSBB, non‐selective beta blockers; WBC, white blood cells.

When a multivariable competing risk regression analysis considering LT as a competing event was performed, age (sHR 1.044; 95% CI 1.019–1.069; *p* = 0.001), belonging to partial (sHR 3.636; 95% CI 1.554–8.507; *p* = 0.003) and non‐responder groups (sHR 5.000; 95% CI 2.218–11.269; *p* < 0.001), higher MELD score (sHR 1.074; 95% CI 1.021–1.129; *p* = 0.006) and lower haemoglobin levels (sHR 0.806; 95% CI 0.715–0.909; *p* < 0.001) were independent predictors of 18 month mortality (Figure 3, Panel B).

## 3 Discussion

Long‐term albumin (LTA) on top of diuretics is associated with ascites control in decompensated cirrhosis, but treatment effects vary across patients, underscoring the need to identify who benefits most [3, 13]. This post hoc analysis of the Real‐ANSWER cohort, a multicentre retrospective study assessing the real‐world use of LTA in Italy, provides important information to cover this unmet need.

The main findings can be summarised as follows: (1) after 3 months of LTA treatment, patients can be stratified in responders, partial‐responders and non‐responders according to the response of ascites; (2) these three categories present different trajectories and clinical outcomes at 18 months; (3) based on these results, a conceptual clinical framework can be hypothesised for integrating LTA with other already available treatment options for patients with ascites.

Responders (patients who resolved ascites to grades 0–1) presented the most favourable courses: they were more likely to discontinue LTA for clinical improvement and had the lowest 18 month mortality. It should be noted that a significant proportion of ‘responders’ received LT after the resolution of ascites. Thus, in these patients, the LTA could be continued with the purpose of controlling ascites and lowering the risk of ascites‐related complications until a liver donor becomes available.

Non‐responders and partial responders were very different from responders in terms of trajectories and survival. Interestingly, responders presented with slightly, but significantly higher MELD scores mainly driven by bilirubin and INR, while partial and non‐responders had higher serum creatinine and white blood cell counts. We hypothesise that this different biochemical profile might reflect distinct pathophysiological drivers: predominant synthetic dysfunction in responders versus a predominant circulatory dysfunction and systemic inflammation in non‐responders. The latter may be associated with reduced response to LTA. However, since more specific markers of inflammation or effective arterial blood volume were not available, this interpretation remains speculative and requires validation.

Both partial responders and non‐responders had a relatively high 18 month mortality rate, and only a minority received LT or TIPS. Unfortunately, the retrospective nature of the study did not allow us to collect detailed information on patients who were or were not considered for LT and TIPS, nor the reasons for their exclusion. However, it should be noted that, in our cohort, only a minority of patients (14%) had a favourable benefit/risk ratio for TIPS at baseline based on the recent EASL guidelines [12]. Furthermore, it was observed that patients who died were significantly older, with a median age close to 70 years, and until a few years ago, this age was considered a contraindication for both TIPS and LT [16]. They also presented with significantly more extrahepatic comorbidities, a higher MELD score and lower haemoglobin levels, all of which have been repeatedly associated with a poor prognosis [17, 18]. In particular, anaemia, which is usually multifactorial, resulting from portal hypertension (hypersplenism, occult bleeding), malnutrition and chronic inflammation [19], has been described as a risk factor for hospital readmissions and ACLF in patients with cirrhosis [17, 20, 21], and is also a well‐known marker of frailty and poor outcomes in the general population [22]. Taken together, these data suggest that the relatively high mortality and low LT and TIPS rates observed in partial and non‐responder patients likely reflect, at least in part, their advanced age and associated comorbidities, as also shown by the results of our PSM analysis.

**FIGURE 4:**
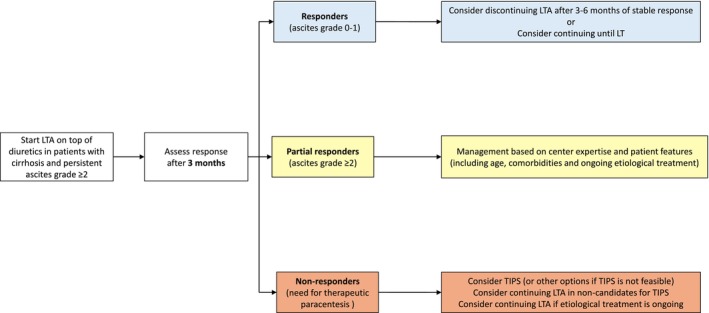
Proposed conceptual clinical framework for the management of patients with cirrhosis and persistent ascites receiving LTA, based on the 3 month prognostic stratification observed in the Real‐ANSWER study. This is intended to support clinical management rather than a validated decision algorithm. (*) Liver transplantation should be considered at any stage. LTA, Long‐term albumin treatment; LT, Liver transplantation; TIPS, Transjugular Intrahepatic Porto‐systemic Shunt.

Based on the results of the current study, together with the already published data [3, 13, 23], we hypothesise a clinical framework to help stratify patients based on the response after 3 months of treatment, potentially facilitating the integration of LTA with other available options (Figure 4).

According to this conceptual framework, LTA can be started in patients with clinically evident non‐complicated ascites (grades 2 and 3) on top of diuretic therapy [6]. Patients with refractory ascites can be also considered for LTA treatment particularly when TIPS is not feasible or the risk/benefit is high and if there is the possibility of a transient or false refractoriness, as it can occur in patients with poor adherence or receiving wrong dosages and combination of diuretics and in those presenting concomitant complications or recovering from a previous complication.

The response to treatment can be assessed after about 3 months. In responder patients who achieve ascites grades 0–1, our data show excellent survival and high transplant rates; thus, continuation of LTA seems rational as a bridge to LT or, in non‐transplant candidates, to maintain ascites control for at least 3–6 months [13]. In these latter cases, if the response persists, an attempt to discontinue LTA could be pursued. On the other side, non‐responder patients still requiring LVP face high mortality. In these patients, the treatment strategy should be re‐assessed and referral for TIPS considered to prevent further clinical deterioration^12^. An exception could be represented by the few patients with an ongoing effective etiological treatment (i.e., antiviral treatment or initial abstinence in patients with alcohol use disorders), which have the possibility to improve liver function leading eventually to ascites resolution and discontinuation of LTA without the need for TIPS insertion [24]. Thus, the response to LTA in these few patients may be assessed at later times. Finally, LTA can be continued when TIPS is contraindicated with the purpose of improving the management of ascites, particularly in those patients showing a reduction in the frequency of paracentesis after starting LTA [13], while palliative approaches can be also considered in non‐transplant candidates.

The partial responder group appears to be more challenging, as these patients suffer from high mortality despite a better control of ascites. In our study population, the advanced age of deceased patients and the high prevalence of comorbidities likely limited their eligibility for LT or TIPS. In such instances, the decision to continue LTA or switch to other strategies should be individualised based on centre expertise and patient features (e.g., comorbidities, diuretic tolerance, ongoing etiologic treatment). In our cohort more than 40% of patients achieved ascites grades 0–1 during follow‐up under LTA, and therefore LTA continuation could be considered. Finally, it is important to underline that, given the observational nature of this study, this proposed strategy is hypothesis‐generating and requires validation in prospective trials to demonstrate its efficacy in improving patient outcomes.

The present study suffers from an inherent limitation represented by the retrospective design, which prevented us from collecting more granular data. In addition, the lack of a control group did not allow us to evaluate the efficacy of LTA compared with other treatments. Besides the fact that this was not the objective of the Real‐ANSWER study [13], a comparison was not possible because LTA has become the standard of care in the participating centres for patients with persisting grades 2–3 ascites over the last decade^6^. Moreover, the criteria used to classify the response of ascites are arbitrary; however, they are based on easily assessable clinical endpoints with different prognostic implications (resolution of ascites and need for paracentesis). Finally, the use of a 3 month landmark analysis introduces a selection bias by excluding about 10% of patients who died or discontinued treatment early. Although we compared the baseline features of patients included and excluded without finding major differences, this does not eliminate the bias.

In conclusion, this real‐life study in patients with decompensated cirrhosis receiving LTA shows that the response of ascites after 3 months from treatment initiation may represent an easy criterion to stratify patients in three groups with different clinical courses and outcomes, helping physicians to personalise ascites management and prioritise treatment choices by integrating LTA with the other options available for each individual patient.

## Author Contributions

E.P., G.I., G.Z., M.B., P.C: study concept and design, interpretation of data, drafting of the manuscript; E.P., G.I., P.T., A.L., S.G., M.T., R.G., D.C., L.L., G.T., S.N., D.B: collection of data; E.P., M.B: analysis of data; E.P., M.B., G.Z., S.P., P.A., P.T., V.C., V.D.M., S.N., P.C: critical revision for important intellectual content.

## Funding

The present study was supported by an ‘Investigator Sponsored Research’ grant by Grifols SA. The Funder had no role in the design and conduct of the study and in the collection, analysis and interpretation of the data, decision to publish or in the preparation of the manuscript.

## Ethics Statement

The study protocol was approved by the local institutional review boards at each participating centre.

## Conflicts of Interest

The following authors disclose conflicts of interest: SP: Plasma Protein Therapeutics Association, Boehringer Ingelheim, Resolution therapeutics (Consultant); Grifols SA and MEDSCAPE (Sponsored lectures). MT: Gilead and Grifols (travel support). GZ: Grifols SA, Octapharma SA (speaking bureau). PA: Biovie (advisory board and patent), CSL Behring (speaker invitation and travel grant), Grifols (speaker invitation), Kedrion (speaker invitation), Biomarin (advisory board), GenFit SA (advisory board). PC: Grifols SA (speaking bureau and advisory boards), Octapharma SA (speaking bureau), CSL Behring (speaking bureau), Gilead (speaking bureau), Takeda (speaking bureau) and Abbvie (speaking bureau). All the other authors disclose no conflicts of interest.

## Supporting information


**Table S1:** Comparison of baseline characteristics between patients included in the study population (*n* = 252) and those excluded due to long‐term albumin (LTA) duration < 3 months (*n* = 30).
**Table S2:** Reasons for discontinuation of long‐term albumin treatment (LTA) in patients excluded from the current analyses due to treatment duration of less than 3 months.
**Table S3:** Comparison of baseline characteristics between Responders and Partial/Non‐Responders in the Propensity Score Matched cohort (*n* = 170).
**Figure S1:** Assessment of covariate balance before and after Propensity Score Matching.
**Figure S2:** Cumulative incidence of liver transplantation (panel A) and mortality (panel B) in the propensity score‐matched cohort.

## Data Availability

The data that support the findings of this study are available from the corresponding author upon reasonable request. The data are not publicly available due to privacy or ethical restrictions.
