# Supplementary material for: Personalised Long‐Term Albumin Treatment Based on Three‐Month Ascites Response in Patients With Decompensated Cirrhosis
Source: Liver Int. 2026 Mar 18;46(4):e70598. doi: 10.1111/liv.70598 (PMC12999546; doi:10.1111/liv.70598)
Supplement: Supplementary file 1 — Table S1: Comparison of baseline characteristics between patients included in the study population (n = 252) and those excluded due to long‐term albumin (LTA) duration < 3 months (n = 30). Table S2: Reasons for discontinuation of long‐term albumin treatment (LTA) in patients excluded from the current analyses due to treatment duration of less than 3 months. Table S3: Comparison of baseline characteristics between Responders and Partial/Non‐Responders in the Propensity Score Matched cohort (n = 170). Figure S1: Assessment of covariate balance before and after Propensity Score Matching. Figure S2: Cumulative incidence of liver transplantation (panel A) and mortality (panel B) in the propensity score‐matched cohort. [file LIV-46-0-s001.docx]

**SUPPLEMENTARY MATERIAL**

**Personalised long-term albumin treatment based on three-month ascites response in patients with decompensated cirrhosis**

**Inclusion and exclusion criteria**

***Inclusion criteria:***

1. patients with diagnosis of cirrhosis based on clinical, laboratory, radiological and/or histological criteria
2. prescription of regular human albumin (HA) infusions for at least 1 month
3. age greater than 18 years

***Exclusion criteria were:***

1. ongoing acute complications of cirrhosis (Hepatorenal syndrome [HRS], spontaneous bacterial peritonitis [SBP] and/or non-SBP-related bacterial infections, grade III/IV hepatic encephalopathy [HE], gastrointestinal bleeding)
2. ongoing acute-on-chronic liver failure (ACLF) defined according to the EASL-CLIF criteria (1)
3. hepatic surgery in the previous 14 days
4. hepatocellular carcinoma (HCC) beyond Milan criteria or other extrahepatic malignancy
5. previous Liver transplant (LT) or other solid organ transplantation
6. previous TIPS
7. severe extrahepatic disease that, according to clinical judgement, may be the predominant factor affecting the patient's prognosis

Reference:

(1) European Association for the Study of the Liver (2023). EASL Clinical Practice Guidelines on acute-on-chronic liver failure. Journal of hepatology, 79(2), 461–491. Https://doi.org/10.1016/j.jhep.2023.04.021

**Supplementary Table 1.** *Comparison of baseline characteristics between patients included in the study population (n=252) and those excluded due to long-term albumin (LTA) duration <3 months (n=30).*

|  | | **Included**  **N=252** | **Excluded**  **N=30** |  |
| --- | --- | --- | --- | --- |
| **Demographic data** | |  |  |  |
|  | Age (years) | 63 (55-69) | 63 (55-74) | 0.526 |
|  | Male sex (n, %) | 175 (69) | 25 (83) | 0.113 |
| **Aetiology of cirrhosis** | |  |  | 0.758 |
|  | Alcohol (n, %) | 99 (39) | 11 (37) |  |
|  | MASLD (n, %) | 37 (15) | 6 (20) |  |
|  | Viral (n, %) | 33 (13) | 4 (13) |  |
|  | Alcohol + viral (n, %) | 27 (11) | 3 (10) |  |
|  | Alcohol + MASLD (n, %) | 29 (12) | 5 (17) |  |
|  | Other (n, %) | 27 (11) | 1 (3) |  |
| **Ascites** | |  |  |  |
|  | Ascites grade |  |  | 0.940 |
|  | Ascites grade 2 (n, %) | 153 (61) | 18 (60) |  |
|  | Ascites grade 3 (n, %) | 99 (39) | 12 (40) |  |
|  | Refractory ascites (n, %) | 72 (29) | 10 (33) |  |
|  | Previous paracentesis within 6 months prior to enrolment (n, %) | 129 (51) | 17 (57) | 0.572 |
|  | - 1-3 paracenteses in last 6 months | 73 (29) | 6 (20) |  |
|  | - ≥ 4 paracenteses in last 6 months | 56 (22) | 7 (23) |  |
| **Medical History** | |  |  |  |
|  | Presence of oesophageal varices (n, %) | 189 (75) | 27 (90) | 0.071 |
|  | Previous overt HE (n, %) | 69 (27) | 11 (37) | 0.286 |
|  | Previous gastrointestinal bleeding (n, %) | 44 (18) | 6 (20) | 0.731 |
|  | Previous spontaneous bacterial peritonitis (n, %) | 29 (12) | 1 (3) | 0.170 |
|  | Previous hepato-renal syndrome (n, %) | 21 (8) | 0 (0) | 0.099 |
|  | HCC within Milan criteria (n, %) | 26 (10) | 3 (10) | 0.957 |
|  | Active alcohol consumption (n, %) | 40 (26) | 4 (21) | 0.653 |
| **Concomitant Medications** | |  |  |  |
|  | Antialdosteronic drugs (mg/day) | 200 (100-300) | 200 (100-200) | 0.299 |
|  | Furosemide (mg/day) | 50 (25-75) | 50 (25-100) | 0.803 |
|  | NSBB (n, %) | 128 (51) | 13 (43) | 0.440 |
|  | Rifaximin for HE prophylaxis | 95 (38) | 12 (40) | 0.806 |
| **Comorbidities** | |  |  |  |
|  | Chronic Heart Disease (n, %) | 43 (17) | 5 (17) | 0.956 |
|  | Chronic kidney disease (n, %) | 39 (16) | 2 (7) | 0.193 |
|  | Chronic lung disease (n, %) | 25 (10) | 2 (7) | 0.567 |
|  | Diabetes (n, %) | 84 (33) | 12 (40) | 0.466 |
| **Laboratory and hemodynamic data at inclusion** | |  |  |  |
|  | WBC (10^9^/L) | 5.3 (3.9-7.2) | 5.9 (3.9-7.2) | 0.779 |
|  | Sodium (mmol/L) | 136 (133-139) | 136 (133-138) | 0.793 |
|  | Bilirubin (mg/dL) | 2.0 (1.2-3.9) | 2.2 (1.0-4.2) | 0.925 |
|  | Creatinine (mg/dL) | 0.9 (0.8-1.2) | 1.0 (0.8-1.2) | 0.690 |
|  | Albumin (g/L) | 31 (27-35) | 32 (29-35) | 0.245 |
|  | INR | 1.4 (1.2-1.6) | 1.4 (1.2-1.6) | 0.956 |
| **Prognostic scores** | |  |  |  |
|  | Child-Pugh score | 9 (7-10) | 9 (8-11) | 0.242 |
|  | MELD score | 14 (11-18) | 14 (11-18) | 0.730 |
|  | MELD-Na score | 18 (15-21) | 17 (14-22) | 0.674 |

*Data are reported by median and interquartile range or absolute frequency and percentage (%) as appropriate.*

*Abbreviation. HCC: hepatocellular carcinoma; HE: hepatic encephalopathy; INR: international normalised ratio; MASLD: metabolic dysfunction-associated steatotic liver disease; MELD: model for end-stage liver disease; MELD-Na: model for end-stage liver disease incorporating serum sodium; NSBB: non-selective beta blockers; WBC: white blood cells.*

**Supplementary Table 2:** *Reasons for discontinuation of long-term albumin treatment (LTA) in patients excluded from the current analyses due to treatment duration of less than 3 months.*

| **Reasons for LTA discontinuation** | **n** |
| --- | --- |
| Stop for clinical improvement (n, %) | 9 (30) |
| Liver Transplant (n, %) | 3 (10) |
| TIPS placement (n, %) | 4 (13) |
| Death (n, %) | 8 (27) |
| Other (n, %) | 6 (20) |
| TOTAL | 30 |

**Supplementary Table 3:** *Comparison of baseline characteristics between Responders and Partial/Non-Responders in the Propensity Score Matched cohort (n=170).*

|  | | **Partial/non-responders**  N=85 | **Responders**  N=85 | **p** |
| --- | --- | --- | --- | --- |
|  | Age (years) | 60.0 ± 9.5 | 60.3 ± 10.3 | 0. 814 |
|  | Any extrahepatic comorbidities (n, %) | 39 (46) | 39 (46) | 1.000 |
|  | Ascites grade 2 (n, %) / grade 3 (n, %) | 66 (78) / 19 (22) | 62 (73) /23 (27) | 0.477 |
|  | Bilirubin (mg/dL) | 2.81 (1.34 - 4.36) | 2.46 (1.50 - 4.50) | 0.903 |
|  | Albumin (g/L) | 30.0 (27.0 - 34.0) | 30.9 (27.0 - 35.0) | 0.935 |
|  | INR | 1.40 (1.23 - 1.60) | 1.49 (1.32 - 1.64) | 0.111 |
|  | Creatinine (mg/dL) | 0.92 (0.73 - 1.20) | 0.87 (0.71 - 1.12) | 0.300 |
|  | Child-Pugh score | 8.9 ±1.7 | 8.9 ±1.7 | 0.957 |
|  | MELD score | 15.4 ± 4.6 | 15.7 ± 4.7 | 0.670 |
|  |  |  |  |  |

Data are presented as mean ± standard deviation (SD), median (interquartile range [IQR]), or number (%), as appropriate. P-values were calculated using Student’s t-test or Mann-Whitney U test for continuous variables and Chi-square test for categorical variables. In the Propensity Score Matching (PSM) analysis responders were matched 1:1 with Partial/Non-Responders based on age, MELD score, ascites grade, and extrahepatic comorbidities using a caliper width of 0.2. *Abbreviations:* MELD, Model for End-Stage Liver Disease; INR, International Normalized Ratio.

**Supplementary Figure 1:** *Assessment of covariate balance before and after Propensity Score Matching. The dot plot displays the standardized percentage bias (Standardized % Bias) for baseline characteristics between Responders and Partial/Non-Responders in the original unmatched cohort (Unmatched, solid dots) and in the propensity score-matched cohort (Matched, crosses). The vertical dashed line at zero represents perfect balance. After matching, all covariates show a significant reduction in bias*

**Supplementary Figure 2**

**Panel A:** *Cumulative incidence function (CIF) of liver transplantation at 18 months in the Propensity Score Matched cohort (n=170).*


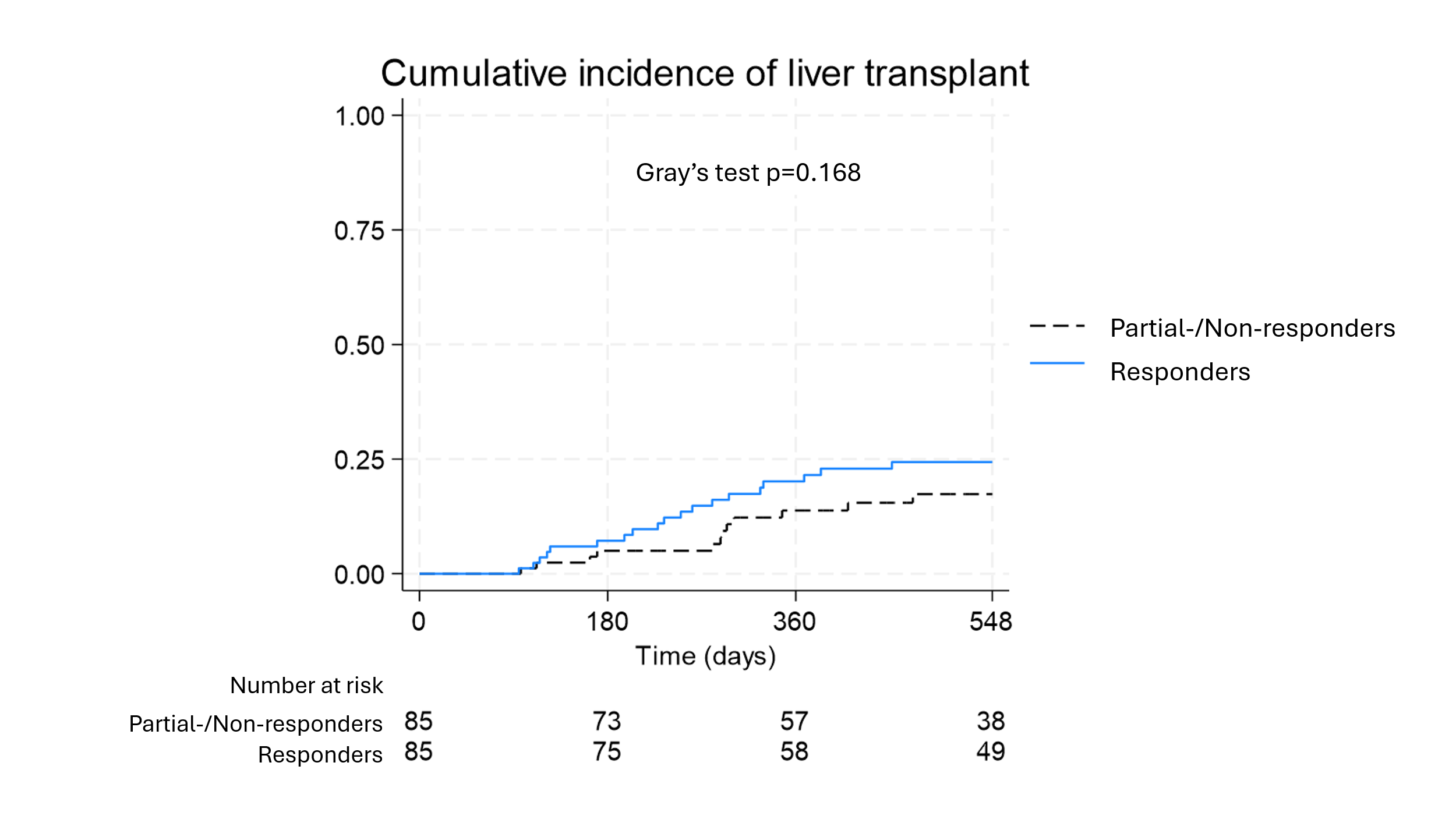


Death was considered as a competing event. The p-value was calculated using Gray’s test. Blue line: Responders; Dashed black line: Partial/Non-Responders.

**Panel B:** *Cumulative incidence function (CIF) of mortality at 18 months in the Propensity Score Matched cohort (n=170).*


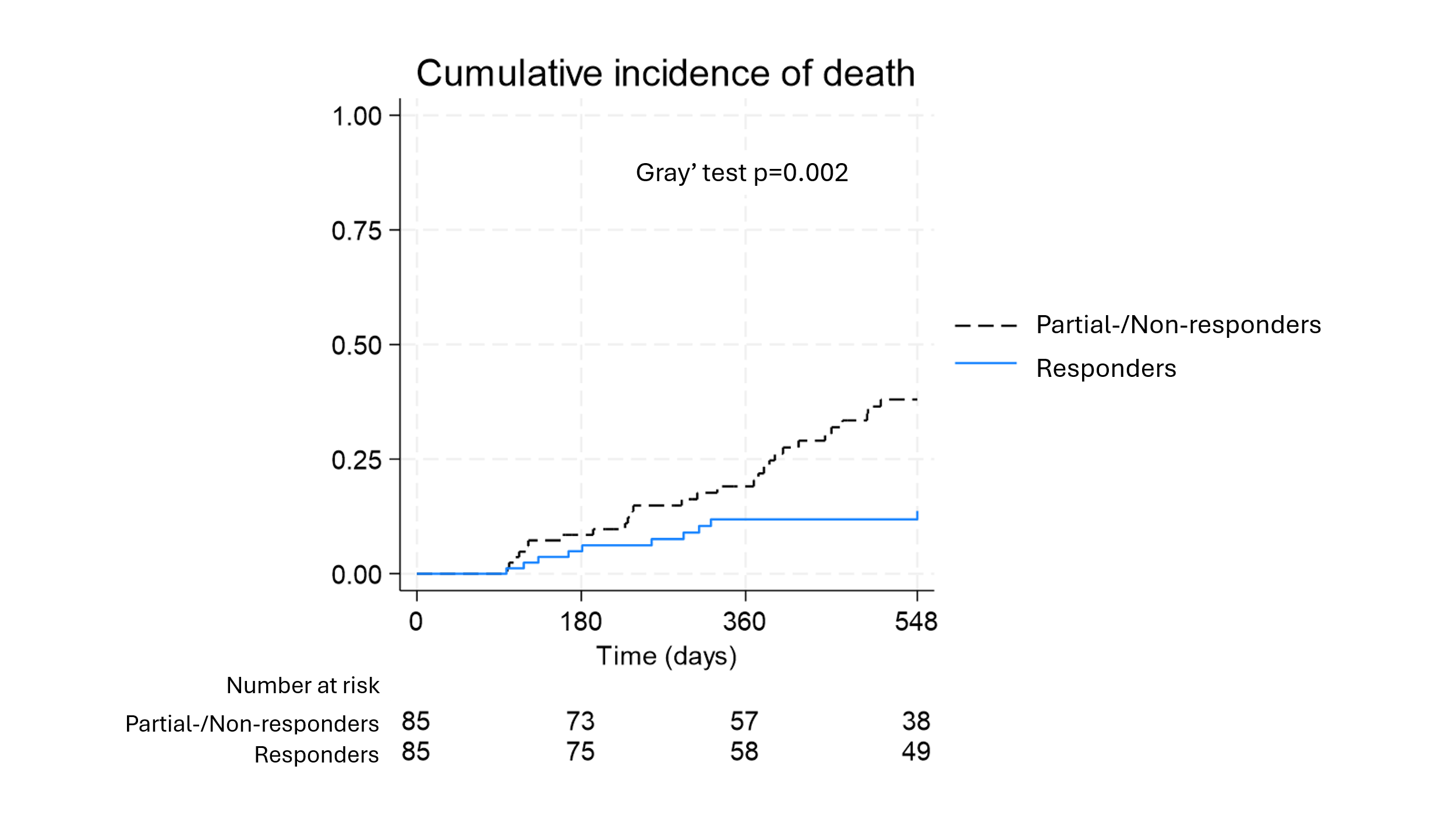


Liver transplantation (LT) was considered as a competing event. The p-value was calculated using Gray’s test. Blue line: Responders; Dashed black line: Partial/Non-Responders.
